# Alginate-Based Biomaterials in Tissue Engineering and Regenerative Medicine

**DOI:** 10.3390/md21030189

**Published:** 2023-03-18

**Authors:** Nima Farshidfar, Siavash Iravani, Rajender S. Varma

**Affiliations:** 1 Orthodontic Research Center, School of Dentistry, Shiraz University of Medical Sciences, Shiraz 71348-14336, Iran; 2 Faculty of Pharmacy and Pharmaceutical Sciences, Isfahan University of Medical Sciences, Isfahan 81746-73461, Iran; 3 Institute for Nanomaterials, Advanced Technologies and Innovation (CxI), Technical University of Liberec (TUL), 1402/2, 461 17 Liberec, Czech Republic

**Keywords:** alginate, biomaterials, hydrogels, scaffolds, tissue engineering, regenerative medicine, biomedical engineering

## Abstract

Today, with the salient advancements of modern and smart technologies related to tissue engineering and regenerative medicine (TE-RM), the use of sustainable and biodegradable materials with biocompatibility and cost-effective advantages have been investigated more than before. Alginate as a naturally occurring anionic polymer can be obtained from brown seaweed to develop a wide variety of composites for TE, drug delivery, wound healing, and cancer therapy. This sustainable and renewable biomaterial displays several fascinating properties such as high biocompatibility, low toxicity, cost-effectiveness, and mild gelation by inserting divalent cations (e.g., Ca^2+^). In this context, challenges still exist in relation to the low solubility and high viscosity of high-molecular weight alginate, high density of intra- and inter-molecular hydrogen bonding, polyelectrolyte nature of the aqueous solution, and a lack of suitable organic solvents. Herein, TE-RM applications of alginate-based materials are deliberated, focusing on current trends, important challenges, and future prospects.

## 1. Introduction

The primary aim of tissue engineering and regenerative medicine (TE-RM) is to create biological substitutes in order to maintain, enhance, or restore damaged tissues/organs [1,2,3,4,5]. It is well known that the application of different types of cells is considered as the most fundamental component of TE-RM strategies [6,7]. However, it has been revealed that following free cell therapy, only a small proportion of cells are viable at the targeted site, and ~90% of them are lost within a few hours after administration [8]. To overcome this challenge, the utilization of cells in combination with biomaterials offers a favorable microenvironment for tissue regeneration [6,9]. Biomaterials are commonly deployed to efficiently transport cells and/or biological factors while also providing an appropriate milieu for cell survival and proliferation [10,11,12,13]. A wide variety of natural and synthetic biomaterials have been introduced that allow the natural deposition of extracellular matrix (ECM) and the regeneration of injured tissues [14].

**Figure 1:**
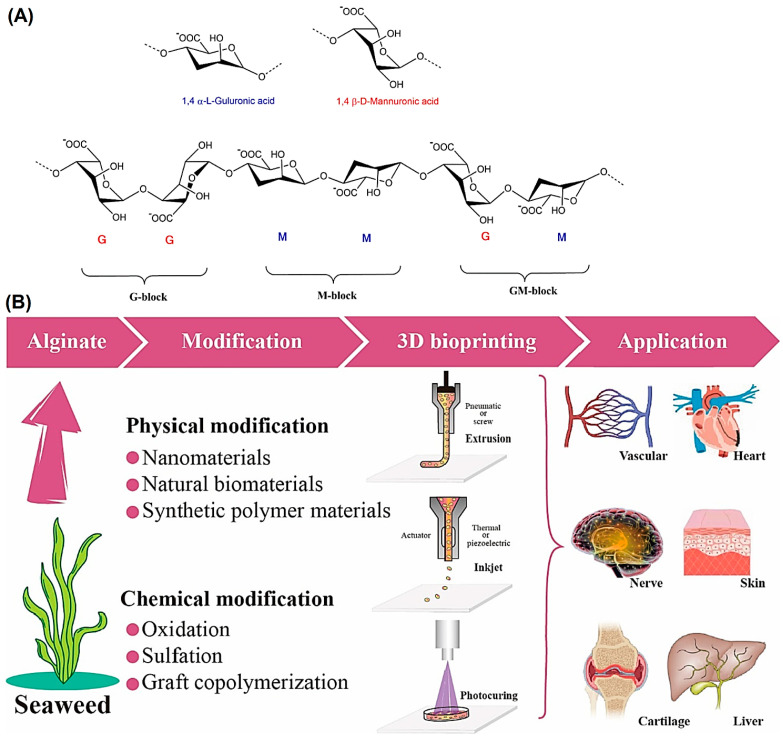
(**A**) The conformation of monomers and block distribution of alginate. Adapted from Ref [18] with permission, Copyright 2020 Springer Nature, licensed under the terms of the Creative Commons Attribution License (CC BY). (**B**) The physical/chemical modification processes, 3D bioprinting techniques, and TE-RM applications of alginate hydrogels. Adapted from Ref. [19] with permission. Copyright 2023 Elsevier.

**Figure 2:**
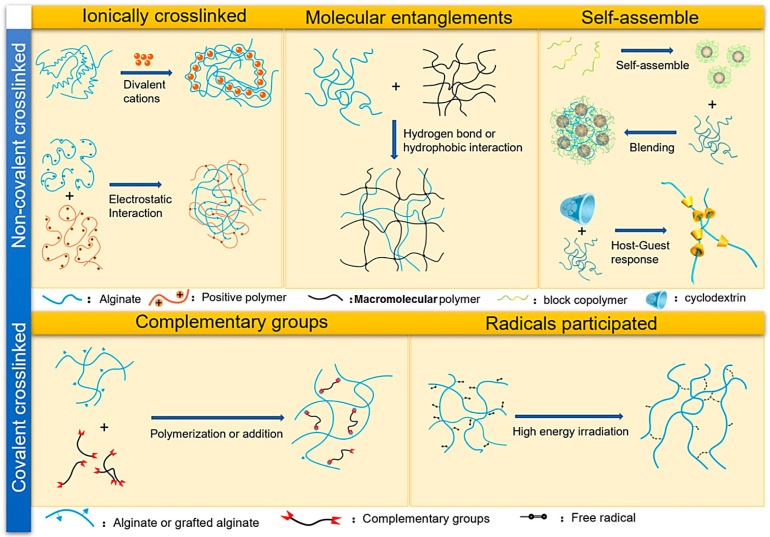
Typical crosslinking approaches to form alginate hydrogels. Adapted from Ref. [23] with permission. Copyright 2020 Elsevier.

Alginate, an anionic and hydrophilic polysaccharide, is one of the most abundant biosynthesized biomaterials worldwide [15]. This natural biomaterial is primarily derived from brown seaweed and bacteria (i.e., Pseudomonas and Azotobacter) [16]. Alginate is also commercially produced using various marine algae such as *Laminaria hyperborea*, *Laminaria digitata*, *Macrocystis pyrifera*, *Ascophyllum nodosum*, *Eclonia maxima*, *Laminaria japonica*, *Lessonia nigrescens*, *Durvillea antarctica*, and *Sargassum* spp. [17]. Alginate contains one thousand blocks of β-d-mannuronic acid (M) and α-l-guluronic acid (G) monomers connected via 1→4 linkage. The blocks are typically composed of three different forms of polymer segments G-block, M-block, and GM-block (Figure 1) [18,19]. Alginate-based biomaterials are developed into different forms such as hydrogels, foams, sponges, fibers, microspheres, and microcapsules using various fabrication techniques [20,21,22]. Nevertheless, alginate hydrogels are of great interest for a wide range of applications, particularly as the supporting matrix or delivery system for tissue repair and regeneration [23]. There are different physicochemical crosslinking approaches (covalent or non-covalent) for fabricating alginate hydrogels that are highly dependent on structure types (Figure 2) [23]. In this context, chelating with divalent cations (Mg^2+^, Ca^2+^, etc.) is the easiest and most cost-effective way to fabricate alginate hydrogels from an aqueous solution under mild conditions [24,25,26,27].

**Figure 3:**
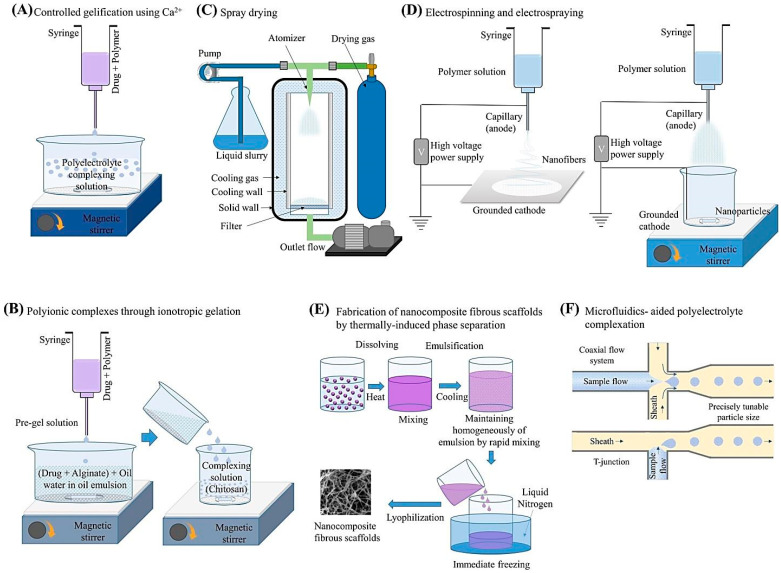
(**A**–**F**) Typical fabrication methods used in synthesizing alginate-based nanomaterials. Adapted from Ref. [17] with permission. Copyright 2020 Elsevier.

Alginate and its hydrogels have been widely employed in TE-RM due to its outstanding properties in terms of hygroscopicity, biocompatibility, biodegradability, non-toxicity, flexibility, and chelating ability [24,28,29]. However, alginate alone has some drawbacks (high viscosity, low solubility, poor degradability, etc.) which hinder its potential for further biomedical applications [30]. To overcome this problem, a wide range of natural or synthetic biomaterials have been incorporated into the alginate structure to enhance its properties and multifunctionality [30]. In addition, modifications of alginate hydrogels with various nanomaterials have recently garnered immense attention, leading to their wide-ranging applications in the biomedical field [17]. There are several fabrication methods deployed for synthesizing alginate-based nanomaterials, the most prominent ones being illustrated in Figure 3. These methods include but are not limited to the controlled gelification, spray drying, electrospinning/electro-spraying, ionotropic gelation, thermally-induced phase separation, microfluidics-aided polyelectrolyte complexation, etc. [17,27,28,30].

Due to the pH-dependent anionic nature of alginate, it has the ability to bind and interact with cationic polyelectrolytes and proteoglycans. These simple electrostatic interactions have been exploited to create delivery systems for cationic drugs and molecules [29]. Alginate hydrogels have also been considered as favorable matrices for the immobilization of various responsive biomaterials and proteins which results in a variety of stimuli-responsive nanosystems for cancer treatment. In addition, alginate-based biomaterials are suitable carriers for gene therapeutic agents owing to their high cell transfection ability for many genes. The networks of alginate-based scaffolds also provide a suitable microenvironment for the cultivation and delivery of therapeutically useful stem cells [31]. For TE-RM, alginate-based scaffolds have been applied in the regeneration of both hard and soft tissues such as skin, bone, cartilage, heart, vascular tissues, etc. [32]. Based on the aforementioned information, alginate, a Food and Drug Administration (FDA)-approved polymer, has become one of the most promising biomaterials for a wide range of TE-RM applications [15,33]. Alginate-based biomaterials have been deployed for TE-RM in the forms of hydrogels/gels, films, fibers, gauzes, foams, wafers, etc. [34]. In this context, their several advantages have been mentioned, including their high porosity and surface area (sponges), inexpensive and easy-to-manufacture nature (gauzes), strong absorption capacity (nanofibers), facilitated cell migration ability (hydrogels), among others [35,36]. The most common technique of alginate gel preparation is ionic crosslinking with multivalent cations such as Ca^2+^, as exemplified in the case of alginate-pectin hydrogel film for diabetic wound healing [37]. Other techniques such as covalent crosslinking (e.g., alginate/chitosan/gelatin hydrogel) [38], enzymatic crosslinking [39], polyelectrolyte crosslinking (e.g., chitosan/alginate hydrogel for bone regeneration) [40], and self-assembly technique (e.g., alginate-based cyclodextrin/azo-polyacrylamide hydrogel) [41], have been introduced. 

Alginate with the advantages of biocompatibility and ease of integration with drugs and cells can be simply processed into 3D scaffolding materials such as hydrogels, foams, sponges, microcapsules, microspheres, and fibers [29,42]. However, pure alginate has limited features (e.g., weak mechanical strength), restricting its future clinical applications; thus, suitable hybridization or combination with other materials can improve its properties for TE-RM. One of the disadvantages of using alginate-based biomaterials is that they have poor cell adhesion properties. In addition, the degradation rate of alginate is variable and should be improved depending on its applications [15]. Remarkably, alginate-based biomaterials have several advantages over chitosan-based biomaterials, such as excellent biocompatibility, biodegradability, and low toxicity. Alginate hydrogels can be simply prepared by crosslinking with divalent cations, offering suitable candidates for TE-RM. In addition, alginate hydrogels offer good mechanical features and can be modified to control their degradation rate. Despite the biocompatibility and biodegradability of chitosan-based biomaterials, chitosan has poor solubility in water at a neutral pH, limiting its applications in TE-RM. On the other hand, chitosan-based hydrogels exhibited the disadvantage of low mechanical resistance, difficulty in controlling the pore size, and uncontrollable dissolution, which restrict their future clinical applications [43]. In addition, chitosan has limited mechanical strength and needs chemical modifications to improve its characteristics. It appears that alginate-based biomaterials have several advantages over chitosan-based biomaterials owing to their high biocompatibility, ease of formation into hydrogels, adjustable degradation rate, and good mechanical features [43,44]. This review endeavored to elaborately discuss the current applications of alginate-based biomaterials in TE-RM, with a focus on recent advancements, important challenges, and future perspectives.

## 2. TE-RM Applications

Several alginate-based composites have been constructed for TE-RM purposes (Table 1). In this context, their biocompatibility and immunogenicity still ought to be systematically evaluated [29,45]. In addition, clinical translational studies and biodegradability improvements still need additional efforts; oxidized alginate displays suitable biodegradability at physiological conditions and can be deployed for targeted drug/cell delivery [45]. For instance, alginate-based hydrogels have been constructed from oxidized alginate, polyethylene glycol (PEG), and carboxymethyl chitosan or gelatin [46]. These hydrogels with a significant degree of crosslinking exhibited the capability of surviving and proliferating mesenchymal stem cells (MSCs), offering them as suitable candidates for injectable self-crosslinking deployment in TE [46]. In another study, thermal-sensitive hydrogels were developed using cystamine-functionalized sodium alginate-g-pluronic F127, showing suitable antibacterial activities and good biocompatibility for long-term cell cultivation [47]. It was revealed that fibroblasts could attach to the hydrogels, which effectively mimic the porous structure of these hydrogels after five days of culture. Such alginate-based composites have been deployed as attractive cellular delivery platforms for versatile TE applications [47]. Overall, the prospects for clinical translation of alginate-based biomaterials are promising because of their biocompatibility and ease of modifications [48]. Some alginate-based composites have been approved by regulatory agencies (such as the FDA) for wound dressings; they have passed clinical assessments and are now available on the market [30,49]. For instance, Kaltostat^®^ (based on calcium alginate or sodium alginate) is one of the commercial wound dressings in the shape of absorbent gel-fiber matrices with fluid contact, thus facilitating atraumatic removal and hemostatic effects as well as helping to control minor bleeding [30]. In another study, alginate-silver wound dressing (Askina^®^ Calgitrol^®^ Ag) was clinically evaluated on patients to compare its activity with the 1% silver sulfadiazine in the outpatient management of partial-thickness burn wounds [50]. Additionally, Emdogain^®^ (Straumann) is a clinically available injectable hydrogel product (porcine enamel matrix derivatives in propylene glycol alginate gel) for the regeneration of periodontal tissue [51]. 

### 2.1. Musculoskeletal TE-RM

**Figure 4:**
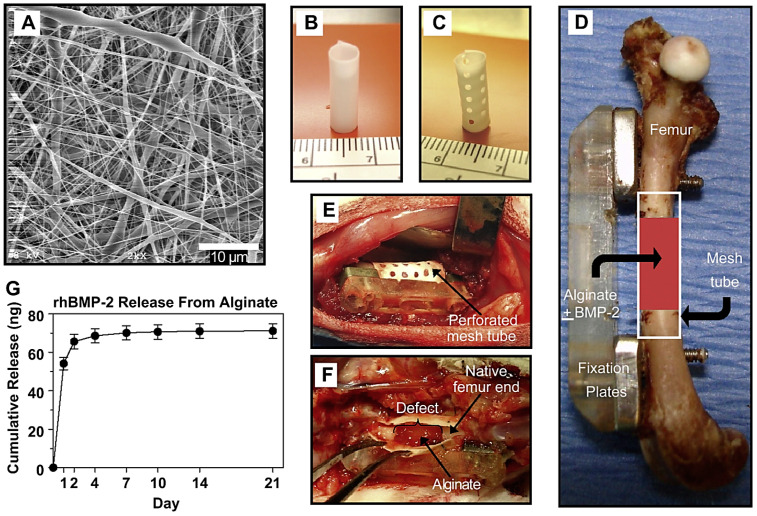
(**A**) Scanning electron microscopy (SEM) image of electrospun nanofiber mesh, showing the smooth and bead-free nano-scaled fibers. (**B**) Hollow tubular implant without perforations constructed from nanofiber meshes. (**C**) Tubular implant with perforations. (**D**) The composite was constructed from an electrospun nanofiber mesh tube applied for repairing the bone defect. (**E**) Picture of the defect after placement of a perforated mesh tube; the alginate inside the tube can be seen through the perforations. (**F**) A specimen was taken down after one week and the mesh tube was cut open. The alginate was still present inside the defect, with a hematoma present at the bone ends. (**G**) The release kinetics of alginate >21 days (in vitro); sustained release of the rhBMP-2 could be detected during the first week. Adapted from Ref. [74] with permission. Copyright 2010 Elsevier.

Scaffolds applied for bone TE and tissue regeneration should have good biocompatibility, osteoconductivity, and biodegradability/bioactivity, along with the promotion of osseointegration as well as stimulation of ingrowth and differentiation of bone [70,71,72]. Different alginate-based composites have been introduced for bone TE applications, including alginate-polymers (e.g., chitosan or PEG), alginate-biosilica, alginate-ceramics, alginate-bioglasses, alginate-bone morphogenetic protein-2, and alginate-proteins (e.g., collagen or gelatin). These composites have shown improved cell adhesion/proliferation, biocompatibility, porosity, mechanical strength, along with alkaline phosphatase (ALP) enhancement, great mineralization, and osteogenic differentiation [28]. Tao et al. [73] prepared polycaprolactone/carboxymethyl chitosan/sodium alginate composite micron-fibers (~2.381 μm) with excellent tensile strength as a scaffold for bone TE through an emulsion electrospinning process, which could facilitate the osteoblast adhesion. The composite could up-regulate the primary expression of osteogenic genes ALP and Runt-related transcription factor 2 (RUNX2), displaying high biocompatibility and osteoinductive potential to osteoblasts. Such composites should be further explored as transplantable scaffolds for treating large-segment bone defects [73]. In another study, an electrospun nanofiber mesh tube was introduced for directing bone regeneration combined with peptide-modified alginate hydrogel injected inside the tube for the controlled release of growth factor (Figure 4). The hybrid delivery system was capable of transferring recombinant bone morphogenetic protein-2 (rhBMP-2) to heal the critically-sized segmental bone defects in a rat model [74]. Despite several advantages of hydrogels, their deployment for repairing bone defects is difficult due to the poor mechanical features and rapid degradation, restricting their bone TE applications. Thus, future explorations ought to be conducted towards the improvement of their mechanical and osteoinductive features through the combination/hybridization with other materials/polymers. In one study, Yan et al. [75] introduced injectable and biodegradable alginate/hydroxyapatite gel scaffolds combined with gelatin microspheres for TE. The addition of hydroxyapatite and gelatin microspheres could improve the mechanical features of these scaffolds, stabilizing the gel network as well as decreasing weight loss, swelling ratio, and gelation time. The alginate-based gel scaffolds with suitable physical activity and bioactive features offer great opportunities for bone TE applications [75].

Alginate-based chitosan hybrid structures were introduced with suitable supporting capabilities for fibroblast adhesion [76]. Compared to the alginate polymer fibers, the prepared alginate-based chitosan hybrid polymer fibers exhibited excellent tensile strength (>200 MPa), offering enhanced adhesion capacity with fibroblasts. After morphologic assessments, it was indicated that the dense fibers of the type I collagen could be generated by the fibroblast in the designed hybrid biomaterials, paving a way for the construction of biomaterial scaffolds with great potential for tendon and ligament TE [76]. Zhou et al. [77] introduced degradable alginate/palygorskite hybrid hydrogels with suitable biocompatibility and robust mechanical features, showing excellent potential for bone defect repair. By increasing the content of palygorskite, the modified hydrogels exhibited improved mechanical features, along with an increased swelling ratio in phosphate-buffered saline (pH = 7.4). The in vitro assessments on bone marrow-derived mesenchymal stem cells (BMSCs) revealed that these composites were cytocompatible after 1, 3, and 7 days [77]. They were loaded with JWH133 (as an agonist of cannabinoid receptor type 2, showing anti-osteoclastogenic and anti-inflammatory effects) to enhance the osteogenic differentiation of rat BMSCs, showing efficient inhibitory effects towards osteoclast generation and the mRNA expression of osteoclast-specific markers. The results of this study indicated that the drug-loading capacity and biocompatibility of alginate-based hydrogels can be improved by suitable modification, paving the way to achieve promising drug carriers against osteoporosis [77]. In addition, 3D-printed scaffolds with desired pore sizes were constructed from sodium alginate and chitosan biomaterials for bone TE and regeneration, exhibiting appropriate capabilities for cell attachment and proliferation. These biocompatible scaffolds with high tensile strength (~0.387 ± 0.015 MPa) exhibited adjustable swelling and degradation manner as well as superb biological properties [78]. 

Despite several natural or synthetic polymers introduced for cartilage TE, alginate-based composites with tunable mechanical properties and easy manufacturing processes have acquired significant attention [79]. A platelet-rich plasma/sodium alginate-based hydrogel was embedded in a porous 3D scaffold of chitosan/chondroitin sulfate/silk fibroin to obtain a hybrid scaffold for cartilage TE applications, showing a uniform distribution of cells and mimicking gel-like cartilage tissue ECM [80]. The matrix exhibited suitable porosity (~77 ± 4.3%) with compressive strength (~0.13 MPa), offering promising structures for cartilage TE. Notably, the introduced cartilage structure exhibited improved metabolic activities, glycosaminoglycan deposition, and collagen type II expression [80]. In addition, a 3D printed scaffold was developed from a double crosslinked alginate hydrogel, in which human umbilical cord MSCs could differentiate into chondrocytes on it after 4 weeks of culturing [81]. After the modification of alginate with L-cysteine and 5-norbornene-2-methylamine, the double crosslinked alginate hydrogels with robust mechanical features similar to natural cartilage were fabricated utilizing CaCl_2_ under ultraviolet light. These hydrogels exhibited long-term stability in Dulbecco’s modified eagle medium (>1 month) with suitable viability for human umbilical cord MSCs. In addition, the expression of chondrogenic genes (e.g., asaggrecan, collagen II, and SRY-box transcription factor-9) could be obtained after culture of human umbilical cord MSCs (4 weeks) in the 3D-printed scaffolds, showing great potential for cartilage repair [81]. 

### 2.2. Cardiovascular TE-RM 

Alginate-based polyurethane elastomers benefiting from the presence of two physical networks of different strengths of soft tertiary ammonium-soft sulfate pairs (as robust ionic bonds) and soft tertiary ammonium-hard carboxylate groups (as the weak bonds) were deployed for vascular TE applications [82]. Notably, considerable toughness/stretchability along with suitable energy dissipation could be obtained due of the existence of sulfate groups, which led to a low Young’s modulus, as well as being endowed with unique self-healing properties. In addition, improved endothelial cell attachment, enhanced anticoagulation performance, and lower platelet adhesion could be obtained, offering these elastomers as attractive candidates for vascular TE applications; the implanted scaffold exhibited low fibrosis and slow biodegradation (within two months) [82]. In addition, alginate dialdehyde was combined with gelatin and MSCs to improve the vascularization capability, along with de novo tissue generation. As a result, alginate dialdehyde-gelatin microcapsules exhibited efficient vascularization by applying an arteriovenous loop tactic, along with enhanced biocompatibility and biodegradability [83]. 

Tubular alginate-based hydrogels combined with collagen type I were fabricated with high mechanical stability and low swelling ratio through an ionotropic gelation process for blood vessel engineering [84]. The alginate solutions were exposed to Ca^2+^-loaded gelatin for controlling the wall thickness of the hydrogels; a second crosslinking phase with barium chloride could prevent their degradation for ~14 days and improve mechanical features by two-fold. It has been revealed that alginate-based hydrogels enriched with collagen were capable of successfully supporting EA.hy926 and MRC-5 cells’ growth and characteristic phenotype, showing promising potential for fabricating freestanding vascular substitutes with controllable features [84]. Additionally, stem cell implantation tactics with promising potentials for the treatment of myocardial infarction (MI) still have some drawbacks, such as low retention and survival, limiting their application due to the reactive oxygen species (ROS) microenvironment after MI [85]. Hao et al. [85] incorporated fullerenol nanoparticles into alginate hydrogel to obtain an injectable cell delivery vehicle with antioxidant properties. The hydrogels with excellent injectability and mechanical strength could successfully scavenge the superoxide anion and hydroxyl radicals; they had no cytotoxicity effects on brown adipose-derived stem cells, thereby suppressing the oxidative stress damage of brown adipose-derived stem cells and improving their survival capacity under an ROS microenvironment through the activation of the extracellular signal-regulated kinase (ERK) and p38 pathways while obstructing the c-Jun N-terminal kinase (JNK) pathway. These fullerenol/alginate hydrogels could effectively reduce the ROS level in the MI region, improving the retention and survival of implanted brown adipose-derived stem cells and inducing the angiogenesis to stimulate the recovery of cardiac functions [85]. 

### 2.3. Neural TE-RM 

Alginate-polyvinyl alcohol hydrogel was deployed as a supportive biomaterial for 3D neural cell cultures, showing stiffness quite similar to brain tissue; neuronal dispersal and 3D network generation could be improved inside the softest hydrogels [86]. In addition, alginate-based hydrogels with a highly anisotropic capillary structure were evaluated for an axon outgrowth assay (in vitro) and healing of spinal cord lesions (in vivo) [87]. Accordingly, the alginate-based scaffolds provoked the regrowth of a vastly oriented linear axon and targeted the re-innervation of a neuron. Notably, alginate-based hydrogels were joined to the spinal cord parenchyma without noticeable inflammatory reactions after implantation into acute cervical spinal cord lesions in adult rats, maintaining their anisotropic structure; they could also stimulate the directed regeneration of axons across the artificial scaffolds. Adult neural progenitor cells with stimulatory effects towards cell-contact-mediated axon regeneration have been seeded into alginate-based hydrogels, improving the regenerative potential of the artificial growth-supportive matrices. Such alginate-based hydrogels can be deployed for inducing directed nerve regrowth after spinal cord injuries [87].

### 2.4. Corneal TE-RM

**Figure 5:**
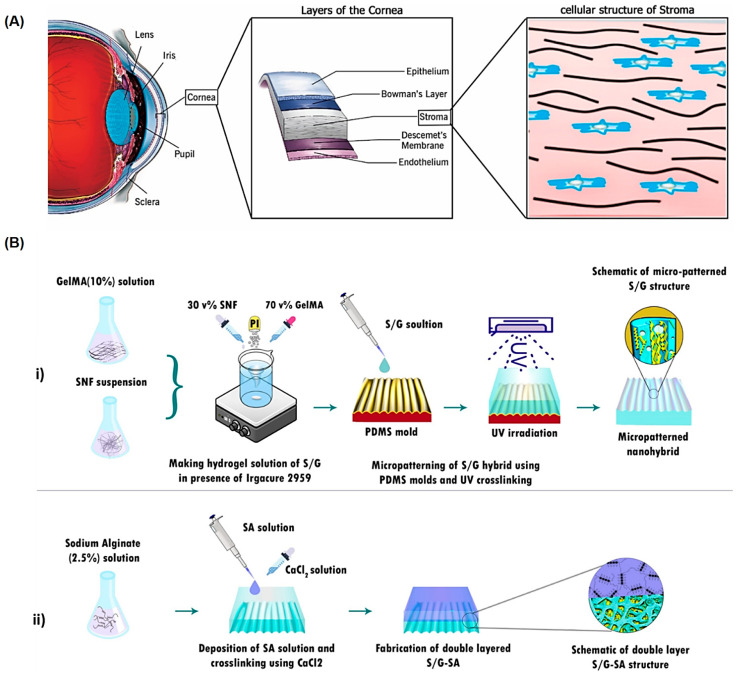
(**A**) The representative corneal stroma structure (layers of the cornea and cellular structure of stroma). (**B**) The preparative process of (**i**) micro-patterned silk nanofibril (SNF) incorporated gelatin methacrylate (S/G) composite films through a micro-molding technique, under ultraviolet light, and (**ii**) double-layer micro-patterned S/G-sodium alginate (SA) film, after crosslinking of SA using CaCl_2_ solution. GelMA: gelatin methacrylate. Adapted from Ref. [89] with permission. Copyright 2021 Elsevier.

Alginates with biocompatibility, non-toxicity, and biodegradability can be deployed for corneal TE-RM, but more elaborative studies are still required to overcome the challenges pertaining to the optimal manufacturing and processing conditions for obtaining alginate-based composites with specific properties [88]. A micro-patterned bioadhesive film with a double-layered structure was developed using silk nanofibrils and gelatin methacrylate-alginate, leading to the sustained release of ascorbic acid for corneal regeneration as well as stimulation of the attachment, alignment, and proliferation of corneal stroma cells (Figure 5) [89]. This hybrid composite film could facilitate the adhesion and orientation of corneal stroma cells, showing suitable mechanical robustness and light transmission owing to the presence of the silk nanofibril/gelatin methacrylate layer; an alginate layer could provide robust adhesion to the tissue. The evaluations indicated that 90% of cells aligned at an angle of 0–20° to the perpendicular axis, signifying the significance of surface micro-patterning to mimic the morphology of the corneal stroma tissue. After accomplishing the optimization process on micro-patterned film, the tensile features near those of native tissue along with the excellent transparency that could be obtained, rendered this composite film suitable for stroma TE-RM [89]. In addition, the alginate-chitosan hydrogel was introduced for limbal stem cells transplantation (in situ), inducing corneal reconstruction after corneal alkali burns [90]. Accordingly, the highly transparent hydrogel with good biocompatibility and low cytotoxicity could be shaped on the wound surface through self-crosslinking with no need for chemical crosslinking components. The hydrogel could significantly improve epithelial reconstruction, leading to rapid and efficient corneal wound healing [90]. It appears that several challenges still exist to specifically control the mechanical and degradation properties of these alginate-based composites for corneal TE-RM purposes [88]. The blend of alginate hydrogels with the protein derived from the ECM (e.g., gelatin and collagen) could provide an improvement in cell adherence, proliferation, and viability in alginate networks. At the post-crosslinking stages, the utilization of suitable chelating agents along with the employment of oxidized alginate form could help improve the biodegradability, drug kinetic release, and functionality of the final alginate-based TE composites [88]. 

### 2.5. Skin TE-RM and Wound Healing

Various types of alginate-based materials have been introduced for wound healing and dressings, including hydrogels/gels, films, fibers, gauzes, foams, wafers, etc. [91]. For instance, vitamin E-loaded hydrogels with biodegradability and high porosity (~89.2 ± 12.5%) were developed using alginate and chitosan for skin TE-RM, showing improved healing properties. As a result, the hydrogel-based dressings exhibited promising potential to treat skin injuries in the clinic [92]. Cai et al. [93] introduced a high-water-absorbing calcium alginate fibrous scaffold prepared after microfluidic spinning and centrifugal reprocessing. The scaffolds with biocompatibility could mimic the ECM, offering promising candidates for the healing of chronic wounds and wound dressings [93]. In another study, a hydroxylated lecithin complexed iodine/carboxymethyl chitosan/sodium alginate composite membrane was prepared using a microwave drying process, which displayed high contents of activated iodine (as antibacterial agents) as well as improved mechanical and swelling properties [94]. These membranes with suitable water vapor permeability exhibited pH-controllable iodine release and high antibacterial effects, which could be deployed for treating chronic wounds and repairing open trauma infections [94].

**Figure 6:**
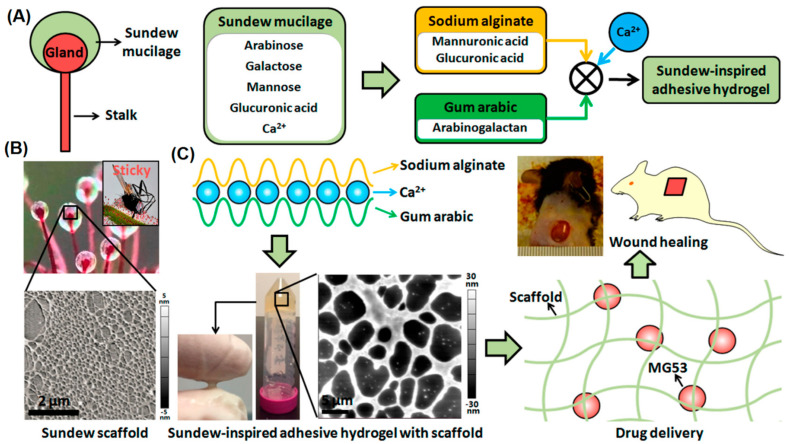
(**A**) The preparative process of bioinspired alginate/gum Arabic hydrogels with sustained drug delivery behavior for chronic wound healing. (**B**) Atomic force microscopy (AFM) topographic micrograph of the sundew mucilage. (**C**) The hydrogels were generated by Ca^2+^-dependent crosslinking between sodium alginate and gum Arabic. Adapted from Ref. [96] with permission. Copyright 2017 American Chemical Society.

Different adhesive peptides or natural/synthetic polymers have been incorporated into alginate fibers to provide desired physicochemical properties and functionality [27]. 3D alginate-based composites were constructed for wound healing applications with high stability and porous architectures [95]. Accordingly, the utilization of the freeze-dried gel of Aloe vera could accelerate the water uptake ability of these scaffolds and also the employed aqueous leaf extracts of *Moringa oleifera* could provide suitable antioxidant, anti-inflammatory, and antimicrobial effects. The scaffolds containing plant extracts and Ca-alginate-polyethylene glycol (PEG)-methyl ether methacrylate exhibited an enhanced cell proliferation capability [95]. In addition, bioinspired alginate/gum Arabic hydrogels were constructed to transfer mitsugumin protein for cell membrane treatment and chronic wound healing (Figure 6) [96]. The introduced sundew-inspired hydrogel exhibited biphasic-kinetics release behavior, facilitating rapid delivery of mitsugumin 53 to improve the re-epithelization procedure of the wounds and sustained release of the protein to treat chronic wounds. Such hydrogels with tunable structures and unique physicochemical features can be deployed as promising delivery vehicles for the healing of chronic wounds [96]. 

### 2.6. Dental TE-RM

Today, studies have focused on using alginate-based biomaterials in dental TE-RM; alginate hydrogels with non-toxicity can be deployed for directing the differentiation of dental-derived stem cells [97,98]. In addition, injectable hydrogels can be deployed as carriers with excellent potential to incorporate cells or growth factors for dental tissue regeneration [99]. In one study, self-crosslinkable hydrogels were constructed from oxidized alginate and carboxymethyl chitosan as cell carriers for dental enamel regeneration (in vitro), showing suitable self-healing property. These hydrogels also exhibited antibacterial activities against *Streptococcus mutans* and *Streptococcus sobrinus*. After in vitro enamel regeneration studies, it was revealed that the dental epithelial cell line, HAT-7, had high cell viability in the injected hydrogels [99]. In addition, an injectable, non-toxic, and biodegradable scaffold was developed using oxidized alginate microbeads, which encapsulated periodontal ligament and gingival MSCs [97]. Compared to the control group, these alginate hydrogels were capable of directing the differentiation of these stem cells to osteogenic and adipogenic tissues (in vitro); these encapsulated stem cells could be viable (in vitro) as well as osteo- and adipo-differentiated after four weeks of culturing in the induction media. Remarkably, the alginate hydrogels had a tunable chemistry and degradation rate, and their degradation profile and swelling kinetics significantly depended on the degree of oxidation [97]. 

Due to their biocompatibility and biodegradability properties, alginate-based scaffolds are promising candidates in dental TE-RM, especially after suitable modification with definite ligands or combined with other substances. An alginate/hydroxyapatite-based nanocomposite scaffold was constructed for bone TE to enhance dental pulp biomineralization and differentiation. As a result, human dental pulp stem cells expressed osteogenic differentiation-related markers and promoted calcium deposition and biomineralization after growing onto the scaffold [100]. Zhu et al. [101] reported that after the combination of alginate with other materials, self-adhesive hydrogels could be designed for dental pulp regeneration. A calcium alginate hydrogel was combined with dental pulp stem cells and fibroblast growth factor 21 for promoting recovery after spinal cord hemi-section in mice through the regulation of apoptosis and autophagy, offering potential for hemi-section spinal cord injury treatment [101]. Liang et al. [102] introduced core-shell microcapsules constructed from gelatin methacryloyl-alginate for the encapsulation of both human dental pulp stem cells and human umbilical vein endothelial cells. Remarkably, human umbilical vein endothelial cells could promote the osteo/odontogenic differentiation of human dental pulp stem cells in microcapsules. In vivo assessments revealed the improved micro-vessel generation and pulp-like tissue regeneration, offering promising candidates for pre-vascularization micro-tissue formation as well as endodontic regeneration and TE purposes [102].

## 3. Conclusions and Perspectives 

Alginate-based biomaterials have been widely exploited for drug/gene delivery, cancer theranostics, antimicrobial agents, wound dressing/healing, and tissue regeneration applications because of their biocompatibility, cost-effectiveness, and ease of processing/functionalization, among others. Remarkably, alginates can be considered as promising biomaterials for bioinks due to their good biosafety and rapid ionic gelation. However, ionically crosslinked alginate hydrogels have the disadvantages of insufficient mechanical properties and long-term stability owing to ion exchange. Furthermore, alginate-based scaffolds still require additional improvements pertaining to their biodegradability, stability, and mechanical properties before they can be routinely applied in clinical stages; these scaffolds should provide suitable structures with optimal cellular activities. In this context, alginate-based hydrogels comprised of collagen or agarose have shown excellent potential as scaffolds for TE-RM due to their good biocompatibility/biodegradability along with their similarity to the natural ECM; thus, offering 3D support for cellular growth and tissue regeneration. Alginate-based biomaterials can also be deployed for the delivery of drugs/therapeutic agents with sustained release behavior, showing improved biodistribution/dissolution rate, bioavailability, and targeting properties. The delivery systems that are composed of these biomaterials have shown improved stability in the acidic environments of physiological fluids. However, systematic in vivo and clinical translational studies as well as biosafety/biocompatibility assessments are still warranted for evaluating their clinical efficacy. Remarkably, finding the optimal manufacturing conditions focusing on solubility, reactivity, and characterizations can help to obtain efficient alginate-based composites with TE-RM applications. Overall, the renewable and sustainable biomaterials can be utilized in hybridization or modification processes to design next-generation alginate-based composites with multi-functionalities. 

## Figures and Tables

**Table 1:** Some selected examples of alginate-based composites for versatile TE-RM applications.

Composites	Applications	Advantages/Properties	Ref
Bioactive transforming growth factor beta 1 (TGF-β1)/hydroxyapatite alginate-based scaffolds	Osteochondral tissue repair	- Proper mechanical features and resorbability - Highly porous osteochondral graft with bioactivity - Suitable cell adhesion and cell-material interaction for up to seven days - No significant evidence of inflammatory reactions (in vivo studies)	[52]
Composite of oxidized alginate, gelatin, and tricalcium phosphate	Bone TE and regeneration	- Good biocompatibility (in vitro) - The composite exhibited potentials as injectable systems for TE	[53]
Oxidized alginate-gelatin-biphasic calcium phosphate composite hydrogels	Bone TE and regeneration	- Hydrogels with good porosity and excellent biocompatibility - Controllable gelation and bio-degradation time - Good mechanical strength	[54]
Chitosan-gelatin-alginate-hydroxyapatite scaffold	Bone TE	- Excellent hydrophilicity and biodegradability - Good mechanical stability - Improved cell viability, proliferation rate, adhesion, and maintenance of osteoblastic phenotype	[55]
Self-crosslinked oxidized alginate/gelatin hydrogels	Cartilage regeneration	- Promising adhesive matrix for neo-cartilage generation - Injectable biomimetic scaffolds for cartilage regeneration	[56]
Collagen-alginate three-dimensional (3D) cell printing bioinks	Cartilage TE	- Improved mechanical strength - Improved cell adhesion and proliferation, along with an enhancement in the expression of cartilage-specific genes (e.g., *Sox9*, *Col2al*, and *Acan*)	[57]
Cell-laden methacrylamide gelatin/alginate hydrogels	Cartilage repair	- Improved biocompatibility and mechanical properties - Excellent potential for cartilage TE	[58]
Covalently polysaccharide-based alginate/chitosan hydrogel-embedded alginate microspheres	Soft TE	- Improved mechanical features and stability - Controlled release of bull serum albumin	[59]
Alginate dialdehyde crosslinked gelatin hydrogels	Polyester vascular graft	- Good biodegradability - Nontoxic, hemocompatible, with sufficient efficiency in sealing the pores of the graft - Enhanced adhesion and proliferation of endothelial cells	[60]
Oxidized alginate/hydroxypropyl chitosan hydrogels	Reconstruction of the corneal endothelium	- Nontoxic and biodegradable - The transplanted corneal endothelial cells by alginate-based composite could survive and retain normal morphology	[61]
Self-crosslinked oxidized alginate-gelatin hydrogels	Muscle TE	- High rate of cell proliferation in the hydrogel with oxidized alginate-gelatin weight ratio of 30/70 - Good biodegradability	[62]
Oxidized alginate covalently crosslinked galactosylated chitosan scaffold	Liver TE	- The porosity of scaffolds was ~70% - Good thermal stability and biocompatibility (in vitro)	[63]
Oxidized alginate hydrogels	Corneal dysfunction treatment	- Oxidized hydrogels with increased pore size and decreased stiffness contributed to enhanced cell viability - Hydrogels holding corneal ECM proteins could affect the function of corneal epithelial cells, with beneficial effects on corneal wound healing	[64]
Hyaluronic acid/Na-alginate films	Bioactive wound dressings/healing	- High antibacterial activities - Effective wound dressings to restore the homeostasis of skin tissue	[65]
Chitosan-alginate films	Wound healing	- Improved mechanical properties; high tensile strength - Non-hemolytic and stable in physiological fluids - The incorporation of thymol and beta-carotene could improve the bioactivity of the formulation	[66]
Cefazolin nanoparticles-loaded crosslinked films of sodium alginate and pectin	Wound dressings	- The alginate-based films with 0.5% crosslinking exhibited improved breaking elongation, water absorption and water vapor transmission, and wetting ratio - Efficient treatment of infections, with improved release profiles	[67]
Alginate-carboxymethyl chitosan scaffold	Enamel TE	- The printed scaffolds were significantly porous - The scaffolds exhibited a high degree of printability and structural integrity - Excellent capabilities to stimulate ameloblast differentiation, calcium phosphate deposition, and matrix mineralization (in vitro study)	[68]
Bioinspired alginate hydrogel	Endodontic regeneration	- Sustained release from the scaffolds; enhanced cell proliferation - Vascular endothelial and fibroblast growth factors synergized co-culture of dental pulp stem cells/human umbilical vein endothelial cells within the scaffolds	[69]

## Data Availability

Not applicable.
